# Cyanobacterial Alkanes Modulate Photosynthetic Cyclic Electron Flow to Assist Growth under Cold Stress

**DOI:** 10.1038/srep14894

**Published:** 2015-10-13

**Authors:** Bertram M. Berla, Rajib Saha, Costas D. Maranas, Himadri B. Pakrasi

**Affiliations:** 1 Department of Energy, Environmental, and Chemical Engineering Washington University, St. Louis, MO 63130, USA; 2 Department of Biology, Washington University, St. Louis, MO 63130, USA; 3 Department of Chemical Engineering, The Pennsylvania State University, University Park, PA 16802, USA

## Abstract

All cyanobacterial membranes contain diesel-range C15-C19 hydrocarbons at concentrations similar to chlorophyll. Recently, two universal but mutually exclusive hydrocarbon production pathways in cyanobacteria were discovered. We engineered a mutant of *Synechocystis* sp. PCC 6803 that produces no alkanes, which grew poorly at low temperatures. We analyzed this defect by assessing the redox kinetics of PSI. The mutant exhibited enhanced cyclic electron flow (CEF), especially at low temperature. CEF raises the ATP:NADPH ratio from photosynthesis and balances reductant requirements of biosynthesis with maintaining the redox poise of the electron transport chain. We conducted *in silico* flux balance analysis and showed that growth rate reaches a distinct maximum for an intermediate value of CEF equivalent to recycling 1 electron in 4 from PSI to the plastoquinone pool. Based on this analysis, we conclude that the lack of membrane alkanes causes higher CEF, perhaps for maintenance of redox poise. In turn, increased CEF reduces growth by forcing the cell to use less energy-efficient pathways, lowering the quantum efficiency of photosynthesis. This study highlights the unique and universal role of medium-chain hydrocarbons in cyanobacterial thylakoid membranes: they regulate redox balance and reductant partitioning in these oxygenic photosynthetic cells under stress.

Cyanobacteria are the most ancient group of oxygenic photosynthetic organisms. They have a specialized intracellular thylakoid membrane system that contains components of the photosynthetic apparatus involved in conversion of solar energy to chemical energy with concomitant oxidation of water to molecular oxygen. These membranes universally include alkanes and/or alkenes of 15–19 carbons. Recently, two pathways for production of these metabolites have been discovered^1^,^2^,^3^,^4^. Although these hydrocarbons were identified nearly 50 years ago^5^,^6^ and are produced at molar concentrations similar to chlorophyll *a*, little is known about their physiological role. The two known pathways for cyanobacterial alka(e)ne biosynthesis either reduce and then deformylate fatty acids using a pair of soluble enzymes (ADO-type), or elongate and then decarboxylate fatty acids using a polyketide synthase-like complex (PKS-type). One of these pathways, but never both, is present in all fully sequenced cyanobacteria^7^,^8^. In this study, we have investigated the function of the *n*-heptadecane produced by the unicellular cyanobacterium *Synechocystis* sp. PCC 6803 (hereafter *Synechocystis* 6803). This strain harbors the ADO-type pathway and is easily amenable to genetic manipulation. It was the first photosynthetic organism to have its genome completely sequenced^9^ and is a common model system for studies on photosynthesis as well as synthetic biology and metabolic engineering^10^. Although efforts have been made to overproduce *n*-heptadecane as a biofuel molecule, they have met with limited success^11^,^12^,^13^,^14^. This inability to overproduce a promising biofuel highlights the gap that still exists between the dream of a plug-and-play microbial cell factory and the reality of a dynamically regulated, intricately interconnected, and poorly understood cellular environment. Therefore, it is important to identify the mechanisms underpinning such overproduction resistance to break down these barriers.

The cyanobacterial thylakoid membrane is unique because it houses both an oxygen-evolving photosynthetic apparatus and a full complement of respiratory enzymes^15^,^16^,^17^. This diversity of functions poses both a challenge and an opportunity: it allows these organisms to adapt to a wide range of conditions and maintain balance among diverse metabolic pathways. However, this membrane system must also carefully regulate both its physical composition and its activities across diverse environmental conditions, since any deviation in the redox poise of the electron transport components can lead to metabolic imbalance and oxidative damage. One challenging environmental condition for cyanobacteria that has been well-studied is cold stress. Cyanobacteria modify their membranes in response to cold stress by synthesizing unsaturated lipids that remain fluid^18^,^19^,^20^. Recovery from photoinhibition depends on maintaining the optimal fluidity of the membrane through fatty acid modification^21^. It has also been shown that cold-stress limits the ability of the cyanobacterium, *Synechococcus* sp. PCC 7002, to utilize nitrate, and requires urea as a reduced nitrogen source for optimal growth^22^,^23^.

Figure 1 provides an overview of the principal components of the photosynthetic machinery housed in the thylakoid membrane. This intracellular membrane system exists in nearly all cyanobacterial strains, often occupying most of the cell volume^24^. The components of this membrane are responsible for capturing solar energy in the forms of ATP and NADPH to power carbon fixation as well as the rest of cellular metabolism. It is critical that these energy sources are produced so as to match their consumption. A number of pathways allow the cell to strike such a homeostatic balance while also maintaining the redox poise of all electron transfer components^25^,^26^. Successful forward electron transfer depends critically on maintenance of redox poise for all components, with deviations leading to unintended reactions and oxidative stress. There are two primary pathways for photosynthetic energy production. In the linear electron transport pathway, electrons travel from water to NADP^+^. They are first excited by light at photosystem II (PSII) where water is split and O_2_ is evolved. These excited electrons are then transported by plastoquinone (PQ) inside the thylakoid membrane to the cytochrome b_6_f complex. Next, they are transported by soluble acceptors such as plastocyanin in the thylakoid lumen to PSI, where they are again excited by light before reaching the final acceptors in the cytoplasm, including NADP^+^, nitrate, and others. Along the way, various steps in the pathway are coupled to transport of protons into the thylakoid lumen to power ATP synthesis by an F_1_F_0_ ATP synthase. This ATP synthesis requires 14 protons to generate 3 ATP, unlike those found in most heterotrophs, which require only 12 protons^27^. The second pathway highlighted in Fig. 1 is a cyclic pathway, in which electrons from PSI are returned to the PQ pool. While several alternative cyclic routes have been proposed, the pathway with the highest quantum yield involves transfer of electrons from NADPH to the PQ pool via the NDH-1 complex^28^,^29^. When electrons are recycled in this pathway, no NADPH but more ATP is produced. Thus, it has been suggested that cyclic electron transport pathways are critical for achieving the appropriate balance of ATP and NADPH to power CO_2_ fixation^25^,^26^,^28^. However, these electron transport pathways must also power other cellular processes such as nitrogen assimilation, macromolecule synthesis, and the carbon-concentrating mechanism. In addition to the high-yield NDH pathway, cyanobacteria also include other forms of NDH-1 specialized for roles in the CO_2_-concentrating mechanism^30^ as well as succinate dehydrogenase^15^ that can participate in cyclic electron transport around PSI. Pseudo-cyclic pathways involving PSII and PSI can also supply extra ATP while reducing oxygen instead of NADP^+^^17^,^26^,^31^,^32^. Table 1 gives an overview of the quantum efficiency of alternative electron flow pathways in *Synechocystis* 6803 for ATP and NADPH production. Because of its prominent role as a model system for photosynthesis studies, much more is known about such pathways in *Synechocystis* 6803 as compared with any other cyanobacterium.

Until recently, physiological roles of the ubiquitous alkanes and alkenes found in cyanobacterial membranes had remained elusive. It was recently found that the α-olefins produced by the PKS-type pathway in *Synechococcus* sp. PCC 7002 play a role in cold-tolerance of this cyanobacterium^33^. In the present work, we find a similar phenotype for *n*-heptadecane in *Synechocystis* 6803. We show that membrane alkanes support optimal growth at low temperatures. Beyond this growth phenotype, we show that a strain that does not produce alkanes relies more on cyclic electron transport, especially at low temperatures. We examine this result in the context of a genome-scale metabolic model that we generated previously for this strain^34^. We used flux balance analysis (FBA)^35^ to explore the role of this pathway in helping cyanobacteria respond to environmental stress. We argue that alkanes are critical metabolites at low temperature because they help to maintain the balance of photosynthetic activities in the thylakoid membrane. We hypothesize that in the absence of alkanes, excess cyclic electron transport is required to maintain redox poise and that this excess leads to the observed slow growth of the mutant strain.

## Results

### Mutant construction

We constructed a plasmid vector, pNOalk (Fig. 2A), to replace the ADO-type pathway for *n*-heptadecane biosynthesis in *Synechocystis* 6803 with a kanamycin resistance cassette. After approximately six months and many rounds of patching on kanamycin-containing BG-11 media^36^ (20–40 μg/mL), we confirmed that no detectable wild-type gene copies remained in the mutant strain via PCR (Fig. 2B). *Synechocystis* 6803 has a high and flexible genome copy number and genes that confer a fitness advantage can be maintained in a merodiploid state when replaced by selective markers. Usually most mutant strains fully segregate within 2–3 patchings. However, to obtain the NOalk mutant, even after several months we had to screen several mutant lines to identify one that was fully segregated. We used GC-MS to confirm that the strain did not produce detectable *n*-heptadecane, and regularly reconfirmed this throughout our experiments.

### Growth and n-heptadecane production

*Synechocystis* grows optimally at 30 C^37^. Although the NOalk strain grew at nearly the same rate as the wild type strain at 30 C, its growth was slower than the wild type at 25 C and severely hampered at 20 C (Fig. 3A). Growth of the wild-type strain was only slightly slower at 20 C than at 30 C. At these lower temperatures, the wild type strain also produced approximately twice as much *n*-heptadecane as when grown at 30 C (Fig. 3B). This increased production of *n*-heptadecane at low temperature and poor growth in its absence indicate that *n*-heptadecane plays an important role in cold tolerance.

### Analysis of Photosynthetic Activities

To investigate why the NOalk mutant grows poorly at low temperature, we analyzed the oxidation/reduction kinetics of PSI reaction centers using a Joliot type JTS-10 spectrophotometer. Photo-oxidized PSI reaction centers (P_700_^+^) have much lower absorption of red light than the reduced form (P_700_), so that a decrease in absorbance at 705 nm signifies increased oxidation of P_700_. In the presence of a long actinic flash, significant net oxidation of P_700_ can occur, especially in the presence of inhibitors of photosynthetic electron transfer reactions. Probing the kinetics of light-induced P_700_ oxidation and its subsequent reduction in darkness is a powerful method for studying *in vivo* photosynthetic electron flow, and in particular, cyclic electron transport^38^,^39^. We suspended exponentially growing WT and NOalk cells in fresh BG-11^36^ media to a chlorophyll concentration of 10 μg/mL. We exposed dark-adapted cells to a 5 second pulse of actinic light and measured the oxidation of P_700_, then switched off the light and measured its re-reduction in the dark over 10 seconds (See Fig. 4). We measured these redox kinetics at both 30 C and 20 C and in the presence of the inhibitors DCMU (3-(3,4-dichlorophenyl)-1,1-dimethylurea) and DBMIB (2,5-dibromo-3-methyl-6-isopropyl benzoquinone). While DCMU blocks the activity of PSII and thus linear electron transport, it allows cyclic electron transport around PSI to continue. DBMIB is a quinone analogue that inhibits the cytochrome b_6_f complex and thus disables both linear and cyclic electron transport^40^, as well as respiratory pathways involving the cytochrome b_6_f complex^15^,^16^.

Figure 4A,C show the effects of these inhibitors at 30 C, while 4B and 4D show the kinetics at 20 C. In the absence of inhibitors, the redox kinetics of P_700_ are nearly the same in the WT and NOalk strains, with the oxidation being slightly faster and reduction slightly slower in the mutant. However, in the presence of inhibitors, the kinetics of reduction diverge. The wild-type strain shows a greater change in oxidation at steady state than the mutant in the presence of either DCMU or DBMIB (Fig. 4A,B). To account for this difference and allow easy comparison among conditions, the kinetics of P_700_^+^ re-reduction in the dark (Fig. 4C,D) have been normalized to the maximal oxidation observed within each measurement.

**Figure 1:**
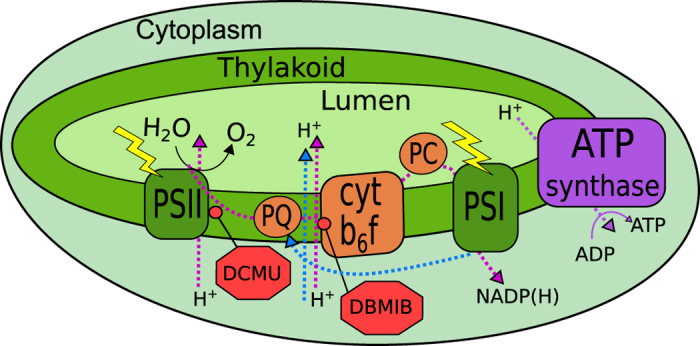
Cartoon of cyanobacterial photosynthetic electron transport pathways. In the linear electron transport pathway (dotted magenta line), light is first absorbed by PSII, then excited electrons are transported inside the membrane by PQ to the cyt b_6_f complex, then through the thylakoid lumen by the PC to PSI. At PSI, electrons are excited by light a second time and then reduce NADP^+^. Along the way, protons are transported to the lumen to power ATP synthesis by the ATP synthase. In the cyclic pathway (dotted blue line), electrons from PSI reenter the PQ pool. Thus, the cyclic pathway produces ATP at the expense of NADPH. Inhibitors used in this study and their sites of inhibition are also indicated in red octagons. DCMU blocks electron transfer from PSII to PQ and DBMIB prevents oxidation of plastoquinone by the cyt b_6_f complex. Cyt b_6_f, cytochrome *b*_*6*_* f* complex; PC, plastocyanin; PQ, plastoquinone; PSI, photosystem I; PSII, photosystem II.

While the re-reduction rates are similar for the WT and NOalk in the absence of inhibitors, the mutant is less sensitive to DCMU as seen by the faster re-reduction of P_700_^+^ with this inhibitor. This difference indicates a lesser reliance on PSII activity to re-reduce P_700_^+^ in the mutant. Therefore, the mutant must be supplying more electrons to P_700_^+^ via cyclic electron transport or respiratory pathways, or else expending fewer electrons via quinol oxidases (See Fig. 1). Assuming that CEF is the major contributor to P_700_^+^ re-reduction, we have used these data to calculate the percentage contribution of cyclic vs. linear electron transport to P_700_^+^ re-reduction^38^ (Fig. 5). The mutant strain at either 20 or 30 C has a greater reliance on cyclic electron transport. However, this difference becomes larger at 20 C, with the cyclic process accounting for nearly 20% of electron flow to P_700_^+^. This would indicate that the cells are recycling approximately 1 electron in 5 in the mutant at 20 C, as opposed to one electron in 11 in the wild type at 20 C and 1 in 17 in the wild type at 30 C.

Another notable feature of the data presented in Fig. 4 is the greater sensitivity of the mutant to DBMIB. At low temperature, the re-reduction half time for the mutant approaches 1 second, which is consistent with back-reaction from ferredoxin, or potentially with the transfer of electrons from NADPH to P_700_^+^ by any number of non-physiological routes. However, for the wild type at either temperature, the re-reduction is ~1.5–2 times faster.

### Flux balance modeling of linear and cyclic electron transport

We earlier developed a genome-scale model of phototrophic metabolism in *Synechocystis* 6803, *i*Syn731^34^. This model includes detailed descriptions of both linear and cyclic electron transport pathways, as well as alternative electron flow pathways, such as via cytochrome oxidase, Mehler reaction, and photorespiration. In this study, we have used the model for flux balance analysis (FBA)^35^ to predict the distribution of all other intracellular fluxes associated with a maximal growth rate under various rates of cyclic electron flow (CEF). Using similar techniques, it was recently found that a range of electron flow pathways allow for robust maintenance of redox poise under a range of environmental conditions in multiple cyanobacterial strains^26^,^32^. We chose to analyze the effect of varied CEF rates because of the observed effect of the NOalk mutation on cyclic electron transport. We found that biomass production is optimal at a recycle rate of 25% (Fig. 6) and decreases approximately linearly both above and below that value albeit with different slopes. At lower CEF rates, ATP production is limiting, while at high recycle rates NADPH production becomes limiting. It is important to stress that the specific recycle rate associated with maximal growth is dependent on environmental conditions such as light intensity and spectral quality, nitrogen source, and the presence of reduced carbon sources.

A closer observation in the *in silico* flux ranges via flux variability analysis^41^ reveals that as the recycle ratio increases, the model predicts expanding feasible ranges of ATP and NADPH production. Based on these results, the model predicts activation of energy inefficient reactions and/or pathways that consume whichever energy source is produced in excess for a particular solution. Because the appropriate ATP:NADPH balance for biomass production must be maintained, these inefficient pathways decrease the quantum efficiency of growth, or biomass produced per photon absorbed.

## Discussion

### Alkane production at low temperature

Similarly to the alkane produced by *Synechocystis* 6803, α-olefins produced by *Synechococcus* 7002 were found to be essential for growth at low temperature. *Synechococcus* 7002 produces both a 19:1 and a 19:2 α-olefin. The level of the 19:2 olefin increased at 22 C, although the total alkene pool decreased^33^. This finding is consistent with the classical paradigm in which membrane unsaturation increases as a response to cold^42^. In contrast, we found that the saturated hydrocarbon *n*-heptadecane accumulated in response to cold. This difference may help to explain why the two pathways for alka(e)ne production are never found to occur together in the same strain^7^, and suggests that their mechanisms of action may be distinct from each other despite the similar phenotypes of these mutants. Alkanes have also been found to accumulate in response to salt and nitrogen stress in some, but not other, cyanobacterial strains^14^.

### P_700_ redox kinetics

*Synechocystis* 6803 exhibits increased cyclic electron flow at low temperature, especially in the NOalk mutant strain. Cyclic electron flow is known to serve diverse roles in autotrophs. Recent evidence suggests that cyclic electron transport in *Chlamydomonas* chloroplasts is regulated directly by the redox state of the cell and acts to prevent over-reduction of the stroma^43^. CEF is also crucial for acclimation of *Arabidopsis thaliana* to fluctuating light^44^. Additionally, CEF can help to maintain the balance of ATP:NADPH required for CO_2_ fixation^45^. Since the thylakoid membrane ATP synthase requires 14 protons to produce 3 ATP, linear electron flow produces ATP and NADPH in a fixed 9:7 ratio and can not provide for the 3:2 ratio of ATP:NADPH to power CO_2_ fixation by the Calvin Cycle^25^ or for the higher ratios required by the rest of cellular metabolism^26^,^32^,^34^. For this reason alone, it has been suggested that PSI would have to recycle approximately 1 electron in 5 to the PQ pool or expend an even greater proportion in pseudo-cyclic electron flow in green algae. The situation in cyanobacteria is somewhat different. Because of the presence of NDH-1 in the cyanobacterial thylakoid membrane^28^, only about 1 electron in 9 would need to be recycled to provide for CO_2_ fixation, although the optimal number depends on the exact mix of nutrients consumed for biomass production. Different nitrogen and carbon sources, especially, will require different inputs of ATP and NADPH for biomass synthesis, as discussed below in the context of our FBA analysis. Additionally, because cyanobacterial thylakoid membranes include a cytochrome c terminal oxidase (CtaI/II)^17^, pseudo-cyclic electron flow around PSII can operate with the same quantum yield of ATP as CEF around PSI, yielding 0.86 ATP per photon (See Table 1). Thus, cyanobacterial thylakoid membranes provide multiple options for the cell to balance ATP and NADPH production under different light quality and quantity, along with the redox state of various electron carriers.

However, the high rates of cyclic electron flow in the absence of alkanes appear to be beyond the range to which this flexible system can adapt while maintaining optimal growth. It remains unclear what exactly the source of this limitation might be. One possibility is that the lack of alkanes reduces membrane fluidity at low temperature, leading to some loss of photosynthetic activity or restriction in electron transport, such as the intramembrane trafficking of reducing equivalents by plastoquinone. In turn, this metabolic inflexibility leads to non-ideal thylakoid reductant partitioning. For example, the lack of alkanes could disable the more efficient NDH-1 CEF pathway or kinetically favor a less efficient pathway, especially at low temperature. This switch to a less efficient pathway could explain the slow growth phenotype and increased CEF requirement. While the mutant displayed a similar effect on its redox kinetics at either ideal or low temperature, the mutant only grew more slowly at low temperature. It seems that with alkanes present, cellular metabolism was able to accommodate the challenge of maintaining a near-maximal growth rate at 25 or 20 C. However without alkanes these low temperatures exceeded the capacity of the cell to adapt (See Fig. 7). Taken together, these data show that the mutant can adapt to the lack of alkanes and low temperatures by increasing CEF, but that such an increase restricts the flexibility of the cell to grow rapidly under diverse environmental conditions.

The slow re-reduction of P_700_^+^ in the NOalk mutant with DBMIB might be explained in several ways. The re-reduction of P_700_^+^ in the presence of DBMIB proceeds primarily by charge recombination within PSI in green algae^46^ and it is likely that the same holds true for cyanobacteria. With a relatively oxidized NADP(H) pool, such charge recombination would proceed more slowly. It is also possible that some of the diverse CEF pathways are less inhibited by DBMIB than others, and that these less-inhibited pathways are less active in the absence of alkanes. Another possibility is that formate dehydrogenase contributes to the re-reduction of P_700_^+^ via cytochrome c553 under these conditions. Because alkane production from fatty acids is a source of formate in the cell, this pathway might be slowed in the mutant. While it is unlikely that this pathway is a major contributor to P_700_^+^ reduction during normal growth, it might have an effect in low temperature.

### Flux Balance Modeling

The FBA derived optimal recycle rate agrees with our observations of cyclic electron flow. We measured a recycle rate of approximately 1 electron in 5 in the mutant at 20 C compared to an optimal recycle ratio of 1 electron in 4 via FBA. While the measured recycle rate for optimal growth was somewhat lower (1 electron in 17 in WT cells at 30 C), it is expected that experimental measurements would be underestimates of the actual rate and that modeling results would overestimate the ideal rate. Both of these discrepancies are caused by the potential activity of cytochrome oxidase. *In vivo*, this activity would lead to an underestimation of CEF rates by intercepting some of the electrons donated to cytochrome b_6_f from reaching PSI. Others have attempted to block cytochrome oxidase activity using potassium cyanide in combination with DCMU for estimation of cyclic electron flow in cyanobacteria, but this inhibitor has given inconsistent results. In some cases, it leads to faster re-reduction of P_700_^+^^38^,^47^, while in other cases the re-reduction is slower^38^,^48^ indicating that this inhibitor has non-target effects. Another possible reason for an underestimation of CEF is that the process is slowed in the presence of DCMU because of a relatively oxidized thylakoid lumen in the mutant. Transfer from PSI electron acceptors to the PQ pool has been regarded as the rate-limiting step in CEF. So, an oxidized pool of PSI acceptors could slow the kinetics of CEF^46^,^49^. *In silico*, by restricting the activity of cytochrome oxidase (and other alternative electron flow pathways) to explore the effect of the dominant cyclic pathway via NDH-1^16^, we eliminated a potential source of ATP and thus required the cyclic pathway to carry a higher flux for ATP production. It should also be noted that an exact match between the simulated environmental conditions in our model and those experienced in our growth studies is difficult to assess, and as we have shown, environmental conditions have a significant impact on cyclic electron flow.

In future work, FBA will serve as a valuable tool for studying the effect of different environmental conditions on cyanobacteria in general and on cyclic electron transport specifically, as we believe that this is a key process that allows photosynthetic organisms to acclimate to a dynamic environment. We have demonstrated here that FBA can serve to analyze phenotypes beyond what is explicitly included in the model. Our model contains no information related to the effects of temperature or a regulatory role of *n*-heptadecane. However, by inputting our observations of increased CEF into the model, we identified a likely secondary outcome of activation of inefficient metabolic cycles for growth. These metabolic reactions would have been impossible to directly measure using available technologies. Thus, an *in silico* approach helped us to understand the mechanistic role of alkanes in a unique way.

## Conclusions

Especially at low temperatures, alkanes modulate reductant partitioning and their absence leads to increased reliance on CEF. We hypothesize that this increase serves to maintain the redox balance of the photosynthetic electron transport chain. In the absence of alkanes, increased CEF represents a less energy-efficient pathway that leads to slower growth at suboptimal temperatures. The low temperature of 20 C used in this study is well within the normal daily range that might be expected in a temperate climate where mid-day temperatures reach or exceed the optimum of 30 C for *Synechocystis* 6803. Redox balance must be tightly controlled by cyanobacteria to avoid redox stress^50^, across the full range of environmental conditions in the organism’s habitat. Thus, for strains living in real environments, alkanes could play a critical role in efficiently using light that is available in cooler morning and evening hours for growth, as well as avoiding redox damage during those potentially stressful periods. It is not unexpected that these hydrocarbons, which are both universal and unique metabolites to the cyanobacterial phylum, should be involved in the challenge of living with light as a primary energy source in a dynamic environment.

The new model we have presented for the role of alkanes in modulating cylic electron flow across environmental conditions challenges previous notions about CEF as an energy generation pathway. This new model should not only enable production of alkanes as biofuels in the future. It also expands our understanding of cyanobacterial photosynthesis and will enable making a wide range of products whose reductant requirements do not exactly match those of normal cyanobacterial growth, including highly reduced, energy dense next generation biofuels like biodiesel and isoprenoids. Beyond any metabolic engineering aims, we have shown both *in vivo* and *in silico* that a range of CEF rates can accommodate growth and may be the best option for an organism under different environmental conditions. We propose that CEF is a flexible redox and nutritional strategy that may be a key way in which cyanobacteria, as well as other photosynthetic organisms, adapt to the demands of generating energy from sunlight under varied environmental conditions.

## Materials and Methods

### Mutant construction

Plasmid pNOalk was constructed via SLIC^51^ and restriction-based cloning. Initially, the fragments immediately upstream and downstream of the *Synechocystis* 6803 genes *sll0208* and *sll0209* encoding aldehyde deformylating oxygenase (ADO) and fatty acyl-ACP reductase (FAR), respectively, were cloned into the pUC118 backbone on either side of a kanamycin-resistance (kan^R^) cassette from pUC4K. Primers were designed using j5 software^52^ and synthesized by IDT (Coralville, IA). Homologous sequences added to assembly fragments at the 5′ ends of primers are in lowercase in Table 2, while sequences binding to template DNA are in uppercase.

**Figure 2:**
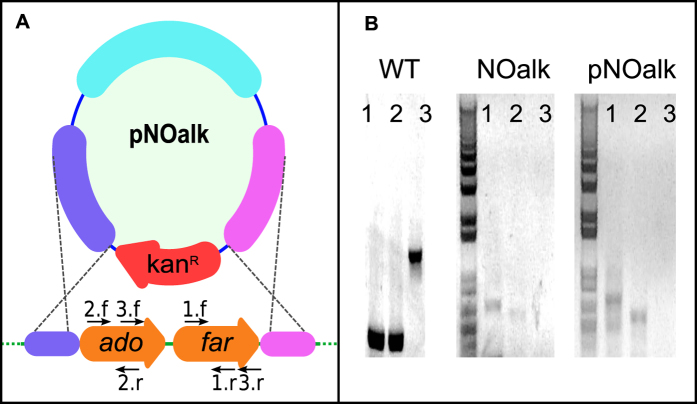
Knockout mutant construction strategy (A) and confirmation by PCR (B). We constructed a plasmid, pNOalk, containing sequences flanking the *ado* and *far* genes for the ADO-type *n*-heptadecane biosynthesis pathway in *Synechocystis* 6803, around a kanamycin resistance cassette (**A**). We confirmed the absence of these genes from the mutant strain via PCR with 3 different primer sets (**B**) using genomic DNA from the wild type or the mutant strain (NOalk), or the plasmid pNOalk as templates. Binding sites of the three primer sets (1,2,3) on the wild-type chromosome are shown in panel (**A**).

**Figure 3:**
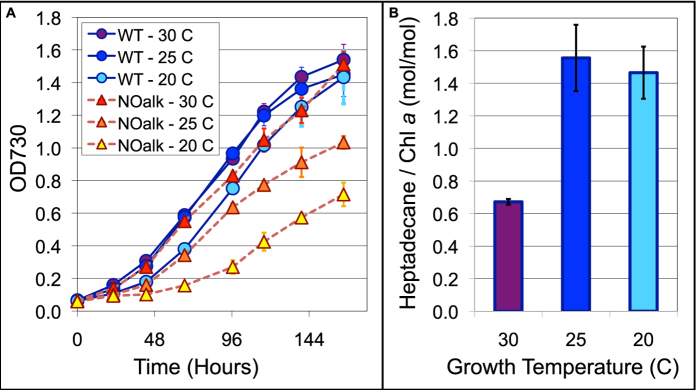
Growth and alkane production by wild type (WT) and noALK strains at various temperatures. (**A**) Cell growth was monitored by measuring OD_730_ daily. (**B**) *n*-Heptadecane was measured via GC-MS and normalized to chlorophyll *a* concentration. Error bars are ± SD for n = 3 for both (**A,B**). Where error bars are not seen, the error is smaller than the symbol shown.

**Figure 4:**
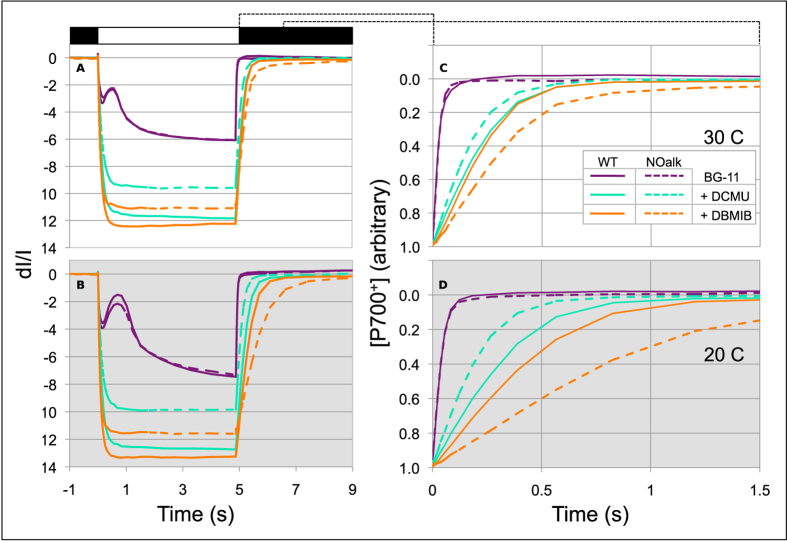
P_700_ redox kinetics for WT and noALK strains at 20 and 30 C. Using a JTS-10 spectrophotometer, cell suspensions were dark-adapted and then exposed to a pulse of orange actinic light (to excite both PSII and PSI) for 5 seconds (white bar above panel (**A**)). The actinic light was then turned off (black bar above panel (**A**)). During this time-course, measuring flashes of 705 nm light probed the redox state of the P_700_ reaction center of PSI. Data were collected from cells that had been grown at 30 C, then measured at 30 C (panel (**A,C**)) or shifted to 20 C before measurement (panel (**B,D**)). Panels C and D show the details of re-reduction of P_700_^+^ in the dark for the experiments in panels A and B, respectively. Inhibitors of linear electron flow (10 μM DCMU, which inhibits transfer from PSII to the quinone pool), and linear + cyclic electron flow (1 μM DBMIB, which blocks cyt b_6_f) were added as indicated. Each trace is an average of 3 independent experiments.

**Figure 5:**
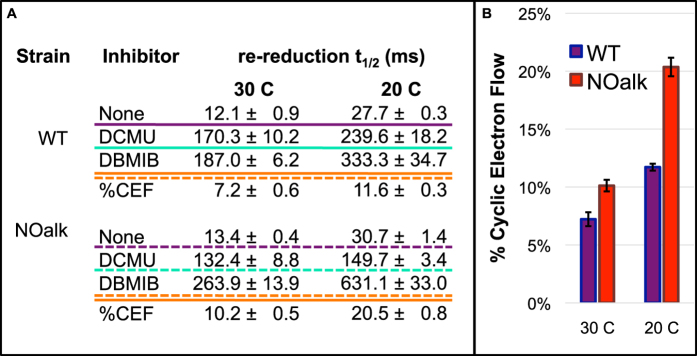
Strain NOalk uses a higher ratio of cyclic:linear electron transport. Panel (**A**) shows the half-times for re-reduction of P_700_^+^ in the dark, calculated from the traces shown in Fig. 4C,D. Panel (**B**) shows the percentage of electron flow to P_700_^+^ that is cyclic, calculated from the data in panel (**A**).

**Figure 6:**
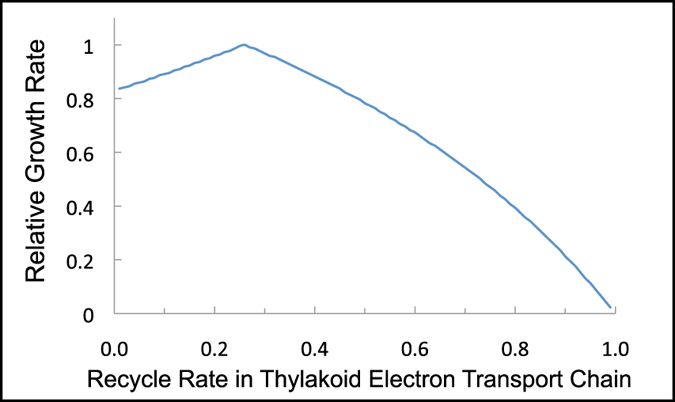
The simulated effect of cyclic electron transport on growth rate using *i*Syn731. We modeled the effect of varying the recycle rate of electrons from PSI to the PQ pool on light-limited growth of *Synechocystis* 6803. The recycle rate was defined as the NDH-1 catalyzed electron flux from NADPH to plastoquinone divided by the electron flux into PSI. These simulations were carried out with alternative electron flow pathways (including succinate dehydrogenase and cytochrome oxidase, see Table 1) restricted to minimal flux. See methods section for further details.

**Figure 7:**
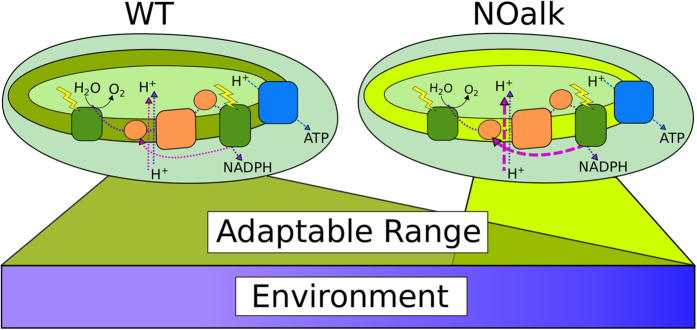
Alkanes impact the adaptability of cyanobacteria to environmental conditions. The lack of alkanes constrains the thylakoid electron transport chain to a higher recycle rate of electrons from PSI to the plastoquinone pool. This inflexibility in reductant partitioning leads to a narrower range of environmental conditions (in particular temperature) in which the strain can grow optimally.

Wild-type *Synechocystis* 6803 was transformed with this plasmid via natural transformation and transformants were isolated on BG-11 media^36^ containing 20 μg/mL of kanamycin. Once colonies appeared, they were restreaked onto fresh media containing 40 μg/mL of kanamycin and the expected insertion was confirmed via colony PCR. Mutant segregation was confirmed by the absence of any detectable PCR fragment originating from the *sll0208* or *sll0209* loci as shown in Fig. 2B.

### Cyanobacterial culture conditions

Cultures were grown in shake flasks at 200 rpm with BG-11 media^36^ under 30 μmoles.m^−2^s^−1^ of white light. For growth experiments, cultures were pre-incubated at their respective growth temperature for 48 hours before being diluted to OD_730_ = 0.05 (~10^7^ cells/mL) in fresh BG-11 media. Cell growth was monitored by measuring OD_730_ daily on a BioTek plate reader (Biotek, Winooski, VT). For *n*-heptadecane analysis, samples were collected after 8 days of growth.

### Extraction and analysis of alkanes

2 mL of culture was pelleted by centrifugation and combined with 1 mL of ethyl acetate and 0.5 mL of 0.1 mm glass beads. Cells were lysed by bead beating (Biospec Products, Bartlesville, OK) for 3 cycles of 1 minute, with 5 minutes rest between cycles. Glass beads and debris were pelleted by centrifugation for 10 minutes at 16,000 × *g*, and then the upper ethyl acetate layer was removed for analysis. Chlorophyll *a* was determined on a DW-2000 spectrophotometer according to the formula [Chl *a*] (μg/mL) = (16.29 × A_665_) − (8.24 × A_652_)^53^. Alkanes were measured on an Agilent 6890 GC-MS fitted with a 12 meter DB5-MS column as previously described^2^ and quantified by comparison with an *n*-heptadecane standard (Sigma-Aldrich, St. Louis, MO).

### Photophysiology experiments

For analysis of P_700_ redox kinetics, cultures were grown as above for 5 days, then diluted 2-fold in fresh media and grown for 24 hours at either 20 C or 30 C. Cells were harvested by centrifugation and resuspended in fresh media to a chlorophyll concentration of 10 μg/mL. Samples were then maintained in the light at their growth temperature until ready for analysis (within several hours). Before each measurement, any required inhibitors were added and then the sample was dark-adapted for 2 minutes. A sub-saturating (130 μmoles.m^−2^s^−1^) pulse of actinic light from an orange LED source was used to illuminate the sample for 5 seconds. During that pulse and for 10 seconds of recovery afterwards, the redox state of P_700_ was monitored by absorption at 705 nm.

### Flux Balance Analysis

Flux balance analysis (FBA)^54^ was carried out using our previously developed *Synechocystis i*Syn731 model^34^ to evaluate maximum biomass production under photosynthetic conditions with various ratios of NDH-1 to PSI activity. The flux distribution for each of these states was inferred using FBA:

### Maximize *V*_*biomass*_

Subject to


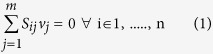



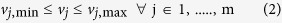



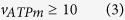



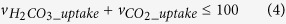



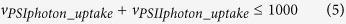



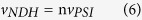



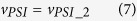



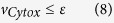


Here, *S*_*ij*_ is the stoichiometric coefficient of metabolite *i* in reaction *j* and *v*_*j*_ is the flux value of reaction *j*. Parameters *v*_*j,min*_ and *v*_*j,max*_ denote the minimum and maximum allowable fluxes for reaction *j*, respectively. *v*_*Biomass*_, *v*_*ATPm*,_ 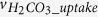, 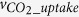, *v*_*PSIphoton_uptake*_, *v*_*PSIIphoton_uptake*_, *v*_*NDH*_, *v*_*PSI*_ and *v*_*PSI_2*_ represent the flux of biomass formation, ATP maintenance, bicarbonate, carbon-di-oxide, PSI photon and PSII photon uptake, NAD(P)H dehydrogenase, PSI (involving reduced/oxidized plastocyanin) and PSI_2 (involving ferro/ferri-cytochrome) reactions, respectively. Note that n is the ratio of NDH-1 activity to PSI activity that is varied in the range of 0.01 and 2 with an increment of 0.01 (equivalent to a recycle rate between 0 and 100%). Following established modeling practices^32^,^34^ with equations (4) and (5), photosynthetic conditions in *Synechocystis* sp. PCC 6803 are represented via setting a ratio of photon uptake (in the form PSI and PSII photon) to carbon uptake (in the form of CO_2_ and H_2_CO_3_) to 1000:100. Also the ATP maintenance requirement of the cell is set to be 10 mmole/g-DW-h and the activity of both the PSI reactions is assumed to be equal^34^. In order to clarify the role of the recycle ratio on the fitness of the organism, the major pseudo-cyclic electron flows involving cytochrome oxidase are downregulated using the constraint as described in equation (8). Here, the value of the parameter **ε** was set to 0.01.

Once we had the values of optimal biomass for various ratios of NDH to PSI activity (as discussed above) from the *Synechocystis i*Syn731 model, flux variability analysis^55^ for the reactions (which participate in electron transport chain in thylakoid lumen and photosynthesis) was performed based on the following formulation:

### Maximize/Minimize V_j_

Subject to


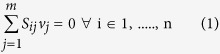



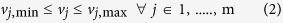



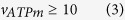



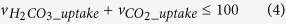



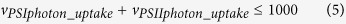



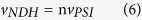



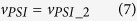



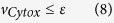



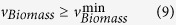


Here, 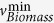 is the minimum level of biomass production. In this case, we fixed it to be the optimal value obtained under a specific NDH to PSI activity ratio in photosynthetic conditions for the *Synechocystis i*Syn731 model.

CPLEX solver (version 12.4, IBM ILOG) was used in the GAMS (version 24.4.4, GAMS Development Corporation) environment for solving the aforementioned optimization models. All computations were carried out on Intel Xeon E5450 Quad-Core 3.0 GHz and Intel Xeon X5675 Six-Core 3.06 GH that are part of the lionxj and lionxf clusters (Intel Xeon E and X type processors and 128 and 128 GB memory, respectively) of High Performance Computing Group of The Pennsylvania State University.

## Additional Information

**How to cite this article**: Berla, B. M. *et al*. Cyanobacterial Alkanes Modulate Photosynthetic Cyclic Electron Flow to Assist Growth under Cold Stress. *Sci. Rep*. **5**, 14894; doi: 10.1038/srep14894 (2015).

## Figures and Tables

**Table 1:** Alternative electron flow pathways and their quantum yields of ATP and NADPH.

Pathway	PSII:PSI activity ratio	ATP quantum yield (h*ν*^−1^)	NADPH quantum yield (h*ν*^-1^)
Linear electron flow	1:1	.32	.25
Cyclic electron flow (NDH-1)	0:1	.86	0
Cyclic electron flow (FQR/SDH/NDH-2/NO_3_ reductase)	0:1	.43	0
Cytochrome oxidase	1:0	.86	0
Mehler reaction/Hydrogenase	1:1	.35	0

Cyclic electron flow pathways around PSI are highlighted in green, and pseudocyclic pathways involving PSII are highlighted in blue.

**Table 2:** Oligonucleotides used in this study.

Primer name	Sequence
sll0208_US_forward	aacagctatgaccatgattacgaatt**CAAAATCTCCGGTCGGGTAAC**
sll0208_US_reverse	gaatatggctcat**AGGGGCGTTGGACTCCTG**
MCS_sll0209_DS_forward	gctcggatccggtaccgtcgactctaga**GCCGACAGGATAGGGCGTG**
sll0209_DS_reverse	tgtaaaacgacggccagtgccaagct**GACAAAAGTGAATGGATGCCCG**
kanR_forward	gtccaacgcccct**ATGAGCCATATTCAACGGGAAAC**
kanR_MCS_reverse	tagagtcgacggtaccggatccgagctc**TTAGAAAAACTCATCGAGCATCAAATG**
1.f	**CGCCCAAGCGGTTGCTGAAGA**
1.r	**GCGCCACAAACCGCTACCGT**
2.f	**AGATGGCGGAACTCTTGCCGGA**
2.r	**ATACCTTGGCGTCCCCCTGCA**
3.f	**TCAGCTACGGCGAAGCCCTCA**
3.r	**CCTAAAGAGCTACTAAAGGGC**

Sequences binding to template DNA are in capital letters, while sequences added to the 5′ end for assembly via SLIC (see methods) are in lowercase.
